# Assessing demographic access to hip replacement surgery in the United Kingdom: a systematic review

**DOI:** 10.1186/s12939-021-01561-9

**Published:** 2021-10-12

**Authors:** Sebastian Ryan-Ndegwa, Reza Zamani, Mohammad Akrami

**Affiliations:** 1 Medical School, College of Medicine and Health, University of Exeter, Exeter, UK; 2 Department of Engineering, College of Engineering, Mathematics, and Physical Sciences, University of Exeter, Exeter, UK

**Keywords:** Hip replacement surgery, Demographic access, United Kingdom, Systematic review

## Abstract

Persisting evidence suggests significant socioeconomic and sociodemographic inequalities in access to medical treatment in the UK. Consequently, a systematic review was undertaken to examine these access inequalities in relation to hip replacement surgery. Database searches were performed using MEDLINE, PubMed and Web of Science. Studies with a focus on surgical need, access, provision and outcome were of interest. Inequalities were explored in the context of sociodemographic characteristics, socioeconomic status (SES), geographical location and hospital-related variables. Only studies in the context of the UK were included. Screening of search and extraction of data were performed and 482 articles were identified in the database search, of which 16 were eligible. Eligible studies consisted of eight cross-sectional studies, seven ecological studies and one longitudinal study. Although socioeconomic inequality has somewhat decreased, lower SES patients and ethnic minority patients demonstrate increased surgical needs, reduced access and poor outcomes. Lower SES and Black minority patients were younger and had more comorbidities. Surgical need increased with age. Women had greater surgical need and provision than men. Geographical inequality had reduced in Scotland, but a north-south divide persists in England. Rural areas received greater provision relative to need, despite increased travel for care. In all, access inequalities remain widespread and policy change driven by research is needed.

## Introduction

A key tenet of the United Kingdom’s National Health Service (NHS) is that access to healthcare should be fair and equal for all [1]. Whilst direct financial barriers to healthcare are mostly absorbed by the NHS in the UK [2], barriers presented by indirect and intangible costs still persist. Studies dating back to 1968 [3] report significant socioeconomic and sociodemographic inequalities in access to medical treatment. These inequalities have endured through time, with those of higher socioeconomic status (SES) still receiving better surgical provisions and outcomes relative to need [1]. Inequalities faced by ethnic minorities need to be acknowledged as they have poorer access relative to White patients [4]. A recent review [5] also highlighted ethnic minority patients’ increased vulnerability to patient safety events, including surgical complications and hospital-acquired infections. Since 2010, government spending on critical social determinants of health has declined by 7% [6]. These reductions have disproportionately impacted the clinical commissioning groups (CCG) responsible for ensuring access to healthcare for the most deprived communities of the UK. Consequently, there is a need to determine which patients face the greatest inequalities to help CCGs plan how to distribute their limited financial resources to those in greatest need. Hip replacement is one of the most frequently performed surgeries in the UK [7], making it a strong case for exploring access inequalities. Hip replacement is cost-effective [8] and improves the quality of life of elderly patients [9]. Given the ageing population of the UK [10], the healthcare burden placed on the NHS is growing. In 2015, it was predicted that the NHS will need to conduct an estimated 439,097 hip replacement surgeries by 2035 [11]. In addition to this figure, almost 100,000 patients are waiting for delayed joint replacement surgery due to COVID-19 [12]. Upper estimates from the British Medical Association suggest that clearing the backlog of elective surgery will cost the NHS £5.4 billion [13]. Delayed hip replacements have a detrimental impact on patients [14], including worse postoperative outcomes, depression [15] and increased reliance on opioid painkillers [16]. The direct and indirect costs associated with delayed surgery increase the financial strain on patients and the NHS [14]. The NHS Long Term Plan [17] aims to improve healthcare access, with an estimated £1 billion spend in areas with the greatest inequalities. Previous financial incentives to promote healthcare equality, such as ‘pay-for-performance’ schemes have been criticised for their inability to tackle ‘pro-rich’ inequalities in hip replacement surgery [18]. Furthermore, evidence suggests that adding this competitive element to healthcare, leads to a fading of ethics in how performance goals are achieved [19]. This ‘ethical fading’ in the pursuit of achieving greater funding, risks exacerbating inequalities in the social determinants of health. By providing a breakdown of factors affecting access to hip replacement surgery, this systematic review aims to provide evidence to inform policy decisions. The objectives will be to: 1) determine which patients experience inequalities in access to hip replacement surgery; 2) determine where these patients are located in the UK and 3) explore other variables that influence the observations, such as differences between hospitals. This review will take a multi-faceted approach by exploring surgical access, need, provision and outcome to achieve a wider picture of which areas drive access inequalities. Inequalities in healthcare access have been reviewed previously [20], but no review has focused explicitly on hip replacement surgery. A PhD thesis [21] containing a systematic review exploring equality in access was identified. However, the scope differed from this review as it included multiple countries and knee replacement surgery.

## Methods

A systematic search of published literature was performed on 4th February 2021. The search strategy followed the Population, Phenomena of Interest and Context (PICo) framework (Table 1).

**Table 1:** Framework for organisation of search strategy

Population (P)	Interest (I)	Context (Co)
Hip replacement recipients and individuals in need of hip replacement surgery	Inequalities in access, need, provision and surgical outcome	I) Impact of sociodemographic variables, socioeconomic status, geographical location and hospital-related variables
		II) Study cohorts and data populations located in the United Kingdom

PICo is explicitly designed [22] for qualitative research and is adapted from the Population, Intervention, Control, Outcome (PICO) framework. Relevant Medical Subject Headings (MeSH) keywords were extracted from the previous review [21] and organised into PICo subheadings. The search was performed in the following databases: MEDLINE, PubMed and Web of Science. The complete search strategy is described Table 9 in Appendix 1.

### Eligibility criteria

Search records were eligible for inclusion provided specific criteria were met (Table 2). Identified articles were reviewed as specified by the Preferred Reporting Items for Systematic Review and Meta-Analyses (PRISMA) 2020 guidelines [23]. Articles were organised, screened and de-duplicated using Rayyan [24], a semi-automated web-tool that assists with exclusion and inclusion decisions while automatically detecting duplicate studies.

**Table 2:** Inclusion and exclusion criteria for included studies

Inclusion criteria	Exclusion criteria
Studies written in English Studies with cohorts in England, Scotland, Wales and Northern Ireland Studies focusing on total hip replacement or with specific data on total hip replacement Publications between 16th December 2005 and 2021^a^ Types of studies: any observational study (cross-sectional studies, ecological studies, case-control studies, longitudinal studies) Peer-reviewed literature	Non-English language studies Studies without UK-based cohorts Studies investigating total hip replacement in combination with other diseases with no specific data or conclusions based on total hip replacement alone Studies published prior to 16th December 2005^a^ Editorials, comments or review articles

^a^To exclude papers covered by previous review, avoiding repetition of analyses/additional overlaps

### Assessment of risk of bias

The risk of bias of the included studies was assessed using a checklist adapted from Mújica-Mota et al. [25] This checklist assesses the impact of selection bias and confounding, two significant challenges.

for observational studies [26]. The checklist questions determine patient characteristics, patient wellbeing, disease severity, detail of hip surgery and hospital-related confounders. The presence of confidence intervals was used to determine whether random error was accounted for in study data. A score was calculated as a sum of the criteria met by each study to facilitate comparison between them.

### Data extraction

Published data from the included studies were extracted under the following categories: study design, year of publication, study population, study timeframe, source of study data and measurement domains. This data is presented in Table 3. These categories were adapted from the study overview presented in a previous paper [21].

**Table 3:** Characteristics of included studies

Ref.	Date	Study design	Study population	Study timeframe	Data Source	Measurement domains
[34]	2009	Ecological study	11,392 English hip replacement patients	SASH data: 1994 to 1995 ELSA data: March 2002 to March 2003	SASH study (local scale) and ELSA study (national scale)	- Age - Gender - Area-based deprivation (2004 English IMD) - Occupational social class - Non-white ethnicity and ethnic mix of residence - BMI Rurality
[2]	2012	Cross-sectional study	54,636 English hip replacement patients	April 2009 to October 2010	PROMs Programme for surgical outcome linked to patient-level HES data	- Age - Gender - Area-based deprivation (2007 English IMD) - Ethnicity - Duration of symptoms Comorbidities
[29]	2009	Cross-sectional study	373 English and Welsh hip replacement patients	2006 to 2007	Internal, patient level data from 16 centres: 13 NHS hospitals, 2 independent sector treatment centres and 1 private hospital	- Age - Gender - Area-based deprivation (2004 English IMD) - Comorbidities - Overall health status Quality of life (EQ-5D)
[27]	2009	Ecological study	406,253 English hip replacement patients	January 1997 to December 2007	Unspecified patient-level hospital data	- Age - Gender - Area-based deprivation (Carstairs index of deprivation) - Rurality of patient residence Provider of care (private, foundation trust, teaching hospital, specialist hospital, traditional NHS)
[1]	2010	Cross-sectional study	n/a	SASH data: 1994 to 1995 ELSA data: March 2002 to March 2003 HES data: 2001/2 financial year	Assessing need: SASH study (local-scale) and ELSA study (national- scale) Assessing provision: Patient-level HES data	- Age - Gender - Area-based deprivation (2004 English IMD) - Ethnic mix of patient residence - Rurality - Distance travelled to receive care - Primary diagnosis Hospital variables (number of hip operations, orthopaedic training centre status, rate of consultants, and operating theatres
[30]	2012	Cross-sectional study	33,709 English hip replacement patients (from 163 separate hospitals)	2001/2 financial year	Patient-level HES data	- Age - Gender - Area-based deprivation (2004 English IMD) - Number of diagnoses at hospital admission - Primary diagnosis Hospital fixed effects
[35]	2015	Ecological study	405,259 English hip-replacement patients	2002 to 2009	ECHO project -patient-level data collected from England, Denmark, Portugal, Slovenia and Spain	- Age - Gender - Area-based deprivation (unspecified index)
[36]	2014	Ecological study	47,069 Scottish hip replacement patients	SMR data: April 1998 to March 2008 ISD data: financial year 2002/3 and 2007/8	Patient-level SMR data and ISD data on NHS-funded hip replacements performed in private sector	- Age - Gender Area-based deprivation (2006 Scottish IMD)
[31]	2007	Cross-sectional study	n/a	Financial year 1991/2 and financial year 2001/2	Patient-level HES data	- Age - Gender Area-based deprivation (Townsend deprivation Z-score)
[32]	2009	Cross-sectional study	n/a	Financial year 2002/3	Patient-level HES data	- Age - Gender - Area level deprivation (2004 English IMD) - Rurality of residence - Ethnic mix of residence Hospital variables (number of hip operations, orthopaedic training centre status, rate of consultants, operating theatres and bed occupancy rates
[4]	2017	Ecological study	293,325 English hip replacement patients	April 2003 to December 2012	Patient-level NJR data linked to HES	- Age - Gender - Area based deprivation (2010 English IMD) - Ethnicity - Primary diagnosis - BMI - ASA score Quality of life (EQ-5D)
[37]	2009	Ecological study	1865 Scottish hip replacement patients	January 1998 to March 2005	Internal, patient-level data from Victoria Hospital, Kirkcaldy	- Age - Gender - Area-based deprivation (2006 Scottish IMD) - BMI - Smoking status - Comorbidities - Surgical complications - Mortality - ASA score of presurgical fitness Quality of life (SF-36)
[28]	2010	Cross-sectional study	282 English hip replacement patients	March 2004 to October 2005	Internal, patient-level data from North Middlesex Hospital, London	- Age - Gender - Area-based deprivation (2004 English IMD) - White ethnicity - ASA score - Surgery duration Morbidity
[39]	2011	Longitudinal study	1312 Scottish hip replacement patients	January 2006 to November 2008	Internal, patient-level data from Royal Infirmary Edinburgh	- Age - Gender - Area-based deprivation (Carstairs index) - BMI - Primary diagnosis - Comorbidities - Surgical complications - 90-day mortality rate Quality of life (SF-12)
[33]	2013	Cross-sectional study	59,680 English hip replacement patients	April 2009 to February 2011	PROMs Programme for surgical outcome linked to patient-level HES data	- Age - Gender - Area-level deprivation (2007 English IMD) - Non-white ethnicity - Primary diagnosis - Comorbidities - General Health Hospital fixed effects
[38]	2011	Ecological study	274,679 English hip replacement patients	2001 to 2008	Unspecified patient-level hospital data	- Age - Gender - Area-based deprivation (2004 English IMD) - Number of diagnoses Hospital activity

## Results

### Search results

**Fig. 1:**
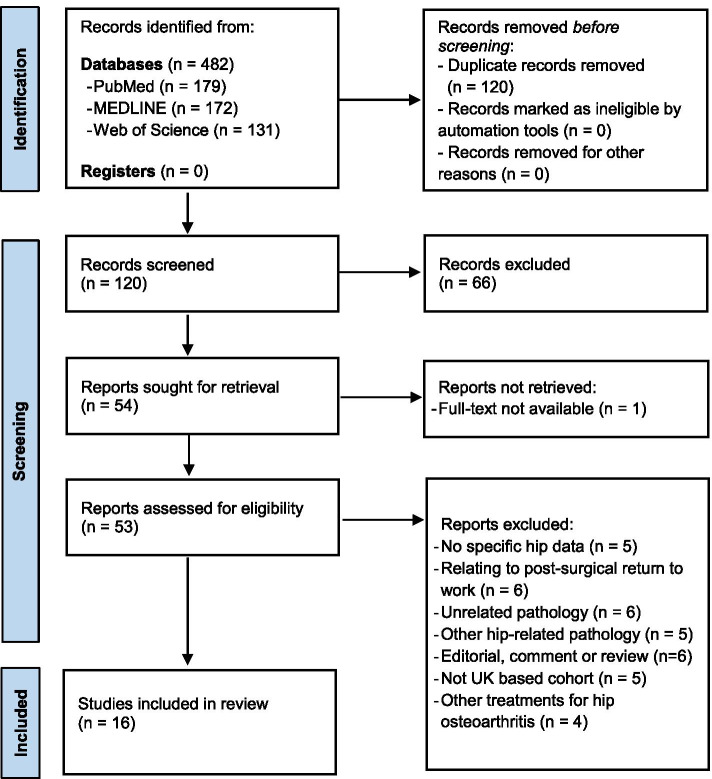
PRISMA flow diagram [adapted from Page et al. [23]]

Over the three databases searched (MEDLINE, PubMed and Web of Science), 482 articles were identified, of which 382 were removed in the deduplication process. With duplicates removed, 120 articles were screened against the inclusion criteria. Sixty-six papers published before December 2005 and those without UK-based cohorts were removed. The remaining 54 articles were screened against the exclusion criteria, using full-text copies, resulting in the removal of a further 38 papers. Reasons for removal are shown Figure 1 in Appendix 2. The most prevalent reasons for removal were studies that discussed an unrelated pathology (*n* = 6), such as shoulder arthroplasty, and studies relating to patients’ postoperative return to work (n = 6). The 16 remaining studies were included in this systematic review.

### Study characteristics

The 16 studies included in this review are of varying characteristics and demographics. Table 3 shows an overview of the study characteristics. The year of publication for the included studies ranged from 2007 to 2016. The largest study [27] explored 406,253 patients over 10 years and the smallest study [28] explored 282 patients over a single year. Of the included studies, 8 were cross-sectional studies [1, 2, 28–33], 7 were ecological studies [4, 27, 34–38] and one was a longitudinal study [39]. The shortest timeframe of the included studies was 1 year [2, 28–30, 32], and the longest was 10 years [27, 31]. The datasets used by studies ranged in age from 1991 [31] to 2012 [4]. In terms of patient-level datasets used, seven studies incorporated Hospital Episode Statistics (HES) data [1, 2, 4, 30–33], an England-wide database of all NHS hospital activity. One study used the Scottish Morbidity Record [36], which provides similar patient-level data to HES, but for Scotland. Four studies used internal hospital data [28, 29, 37, 39] and two studies used unspecified national, patient-level data [27, 38]. Two studies used a combination of local-scale and national-scale, patient-level data from the Somerset and Avon Survey of Health and the English Longitudinal Study of Ageing, respectively [1, 34]. English Indices of Multiple Deprivation (IMD) were used by 10 studies to assess SES [1, 2, 4, 28–30, 32–34, 38], 7 of which used the 2004 edition [1, 28–30, 32, 34, 38], 2 used the 2007 edition [2, 33] and one used the 2010 edition [4]. Scottish IMD (SIMD) were used by two studies, both of which used the 2006 edition [36, 37]. Two studies used the Carstairs Index [27, 39]. One study used the Townsend index [31]. An unknown quintile-based deprivation index was used by one study [35]. A complete list of assessed domains is provided in Table 3.

### Risk of bias

The risk of bias checklist for assessing the quality of the included studies is shown in Table 4, with the number of questions answered by each study for comparison. All studies recorded the age and gender of the study population. Of the 16 studies, only one included data from private hospital admissions [29]. Two reported the distance travelled by patients to receive hip replacement surgery [1, 32]. Three studies had information on the rurality of patients’ residences [1, 27, 32]. Four studies had information on patients’ quality of life [4, 37–39] or their Body Mass Index (BMI) scores [4, 34, 37, 39]. Two studies met the most quality criteria, answering 12 out of 16 questions (75%) [1, 4]. One study met the fewest quality criteria, answering only five questions (31%) [31]. On average, the included studies answered nine questions for quality criteria (56%).

**Table 4:** Risk of bias checklist for assessing study quality

	Study reference															
Question	[34]	[2]	[29]	[27]	[1]	[30]	[35]	[36]	[31]	[32]	[4]	[37]	[28]	[39]	[33]	[38]
Were the following study characteristics reported:																
- *Study population*	●	●	**●**	●		●	●	●			●	●	●	●	●	●
- *Age*	●	●	●	●	●	●	●	●	●	●	●	●	●	●	●	●
- *Gender*	●	●	●	●	●	●	●	●	●	●	●	●	●	●	●	●
- *Ethnicity*	●	●			●					●	●		●		●	
- *Primary diagnosis*		●			●	●	●			●	●	●		●	●	●
- *BMI*	●										●	●		●		
- *Rurality of residence*				●	●					●						
- *Distance travelled for care*					●					●						
Were patient-recorded outcome measures (PROMs) assessed?	●	●	●		●							●		●	●	
Was patient-recorded quality of life assessed?			●								●	●		●		
Was the type of hip surgery reported?			●	●	●	●	●	●	●	●	●	●	●	●	●	●
Was a distinction made between primary and revision surgeries?	●	●		●	●	●	●	●		●	●		●	●	●	●
Were hospital-related factors assessed? (including use of hospital fixed-effects models)					●	●				●	●				●	●
Did the study assess data from more than one hospital?	●	●	●	●	●	●	●	●	●	●	●				●	●
Was data from private hospitals included? (excluding ISTCs)			●													
Were confidence intervals presented with data analysis?	●	●	●	●	●		●	●	●	●	●	●	●		●	
**Outcome score:**	9	9	9	8	12	8	8	7	5	11	12	9	7	9	11	8

Note: ISTC = Independent sector treatment centre; PROM = Patient-reported outcome measure

### Need for hip replacement surgery

Table 5 shows the results for the three included studies that explored the need for hip replacement surgery [2, 29, 34]. Judge et al. [34] reported that 31.9 per 1000 English residents over 50 years old were in need of a hip replacement (CI: 28.4–35.8). Need was greatest for patients living in the most-deprived areas (IMD Q5). A stronger, linear relationship was found between occupational social class and need, with the lowest social class (class V: unskilled) having the greatest surgical need. Neuburger et al. [2] showed that, before surgery, mean Oxford Hip Score (OHS) was 3.6 points lower in the least-deprived patients than the most-deprived (IMD Q5 versus Q1) (CI: 3.4–3.9). Soljak et al. [29] reported a similar trend in OHS, with mean OHS 3.5 points lower in the least-deprived patients than the most-deprived (IMD Q5 versus Q1) (CI: 0.078–0.274). However, adjusting for age, sex, general health, comorbidities and patient-reported quality of life (EuroQol 5-dimension scale [EQ-5D]) lowered the level of significance, increasing the *p*-value from *p* < 0.001 to *p* = 0.02. Neuburger et al. [2] showed the most-deprived patients (Q5) experienced hip problems for a longer duration than the least-deprived (Q1) (CI: 1.03–1.20). Longer-term hip problems were also associated with patients younger than 50 years old compared to patients aged 71 to 80 (CI: 3.90–4.64). Despite this, Judge et al. [34] found that rates of need increased with age, with patients aged over 85 years experiencing the greatest need after adjustment for obesity. Patient BMI scores above 30 (obese) were a strong predictor for surgical need (CI: 1.9–2.8). Women have a greater reported need for surgery than men (CI: 0.6–0.9), with Neuburger et al. [2] reporting a mean presurgical OHS 2.3 points lower than for men. Despite this, women had a lower likelihood of reporting long-term hip problems than men (CI: 0.92–1.00). South Asian and Black patients had lower mean OHS than White patients. South Asian patients had mean OHS 2.7 points lower than White patients and Black patients had mean OHS scores 0.9 points lower. However, when comparing the mean OHS of Black and White patients, the adjusted differences were not statistically significant at the 5% level. Judge et al. [34] found that whilst univariable analyses suggested non-White patients had a greater surgical need, this effect was due to confounding from area-based deprivation and social class. Furthermore, no association was found between the ethnic mix of patients’ residence and surgical need. Neither was any association found between the rurality of patient residence and surgical need.

**Table 5:** Results of studies exploring surgical need

Ref.	Study timeframe	Estimate of surgical need	Socio-demographic domains	Conclusions
[34]	SASH data: 1994 to 1995 ELSA data: March 2002 to March 2003	New Zealand score for joint disease severity (proxy score calculated from SASH and ELSA data)	- Age - Gender - Area-based deprivation (IMD 2004) - Occupational social class - Non-white ethnicity and ethnic mix of residence - BMI - Rurality	-After multivariable adjustment, the rate for hip replacement was 31.9 per 10,000 (95% C.I: 28.4–35.8) Age - Rates of need increased with increasing age, with effect reinforced after controlling for obesity Sex - Rates of need are lower in men than in women (95% CI: 0.6–0.9) Deprivation - Lowest social class have the greatest rates of need, however this association is attenuated by deprivation and obesity - Least deprived patients also have greatest rate of need however effect is not as strong as class Ethnicity - Data suggested non-white patients had greater rate of need, however adjustment for the confounders of deprivation and social class eliminated this - Ethnic mix of patient residence is not associated with rate of need Rurality - No association between rurality and rate of need was found Obesity - BMI scores above 30 were a strong predictor for increased rate of need (95% CI: 1.9–2.8)
[2]	April 2009 to October 2010	Oxford hip score to determine severity of hip condition Patients asked how long their hip problems been present and which comorbidities they have	- Age - Gender - Area-based deprivation (IMD 2007) - Ethnicity - Duration of symptoms - Comorbidities	Deprivation - More deprived patients had lower mean OHS than less deprived with clear gradient across deprivation quintiles: difference between least and most deprived was 3.6 points (95% CI: 3.4–3.9) - More deprived patients had longer-term hip problems with an odds ratio of 1.11 (95% CI: 1.03–1.20) Gender - Women had mean OHS 2.3 points lower than men (95% CI: 2.2–2.5) - Women also less likely to self-report long-term issues with an odds ratio of 0.96 (95% CI:0.92–1.00) Age - Patients who had hip replacement at atypically young or old age had lower mean OHS: OHS score 1.4 points lower between youngest patients (below 51 years) and patients aged 71-80 years (95% CI: 1.1–1.7) - Duration of hip-related issues declined with age with odds ratio of 4.26 between youngest patients and those aged 71-80 (95% CI: 3.90–4.64) Ethnicity - More severe hip condition in South Asian patients (OHS 2.7 lower) and Black patients (OHS 0.9 lower) than White patients – also had longer-term issues with odds ratio of 1.40 - South Asian and Black patients had lower pre-surgery OHS than White patients, with South Asians having lowest mean OHS (2.7 points lower) (95% CI: 1.5–4.0) - Differences between White and Black patients not statistically significant (mean OHS 0.9 points lower)
[29]	2006 to 2007	Oxford hip score to determine severity of hip condition EQ-5D quality of life questionnaire Patients asked how long their hip problems been present, which comorbidities they have, their overall health status and whether they’ve had similar surgery before	- Age - Gender - Area-based deprivation (IMD 2004) - Comorbidities - Overall health status - Quality of life (EQ-5D)	Deprivation - Mean OHS 3.5 points lower in most deprived patients versus least deprived patients- significant relationship (P < 0.001) - Adjustment for age, sex, health status and comorbidities weaken this relationship: increased *p*-values and fall in standardised regression coefficients - After adjusting for quality of life (EQ-5D), regression coefficients fall further, however relationship still remains significant (p = 0.02)

NOTE: SASH = Somerset and Avon Survey of Health; ELSA = English Longitudinal Study of Ageing; IMD = Indices of Multiple Deprivation; BMI = Body mass index; C.I = confidence interval; PROM = Patient-Reported Outcome Measure; HES = Hospital Episode Statistics; OHS = Oxford Hip Score; EQ-5D = EuroQol-5D; NHS = National Health Service

### Access to hip replacement surgery

Table 6 shows the results for the five included studies that explored overall access to surgery [1, 27, 30, 35, 36]. Judge et al. [1] reported 70% less provision relative to need in the lowest SES patients for England (95% confidence interval: 0.30–0.33). Cookson et al. [35] showed that the ratio between provision and need increased by 12% (CI: 1.23–1.35) from 2002 to 2009. Judge et al. [1] reported that for every 1000 English people in need of hip replacement, only 44 will undergo the operation. Cookson et al. [35] report that, the average rate of hip replacement across England, in 2009, was 20.2 per 10,000 people over 35 years of age. When adjusted for age and sex, hip replacement rates were higher in the least-deprived quintile (Q1) than the most-deprived (Q5), with a Q5/Q1 ratio of 1.35 (CI: 1.25–1.45); that is,Q1 patients were 35% more likely to undergo surgery than Q5 patients. Kirkwood et al. [36] reported that while geographical inequality significantly improved in Scotland from 1998 to 2008 (*p* < 0.001), socioeconomic inequality did not change significantly. Judge et al. [1] also noted greater access inequality in the West Midlands, London and the north of England, with patients in the south of England experiencing greater provision relative to need. Increased rurality in England was associated with greater provision relative to need, as were longer road travel times for care. Kirkwood et al. [36] reported that hip replacement rates were significantly lower in the most-deprived SIMD quintile (Q5) than any other quintiles (Q1-4).

**Table 6:** Results of studies exploring general access

Ref.	Study timeframe	Estimate of surgical need	Estimate of surgical provision	Socio-demographic domains	Conclusions
[27]	January 1997 to December 2007	n/a	Waiting time until hip replacement from initial specialist referral to surgery	- Age - Gender - Area-based deprivation (Carstairs index of deprivation) - Rurality of patient residence - Provider of care (private, foundation trust, teaching hospital, specialist hospital, traditional NHS)	- Statistically significant reduction in waiting time with the following successive time periods:1997-2000, 2001-4, and 2005-7 Deprivation - Positive association between deprivation and waiting times in 1997 - From 1997 to 2000, successive increases in deprivation quintile (least deprived ➔ most deprived) were associated with significant increase in waiting time by 1-2 weeks (*P* < 0.001) - From 2001 to 2004, there was large variation in waiting time between deprivation quintiles with middle quintile patients waiting longest - From 2005 to 2007, there was little association between deprivation quintile and waiting time - Waiting times had more of a uniform distribution by 2007 - Variation in waiting time in relation to socioeconomic group decreased over time
[1]	SASH data: 1994 to 1995 ELSA data: March 2002 to March 2003 HES data: 2001/2 financial year	New Zealand score for joint disease severity (proxy score calculated from SASH and ELSA data) Patients scoring below 48/80 on New Zealand score excluded	Admissions data from HES	- Age - Gender - Area-based deprivation (2004 IMD) - Ethnic mix of patient residence - Rurality - Distance travelled to receive care - Primary diagnosis - Hospital variables (number of hip operations, orthopaedic training centre status, rate of consultants, and operating theatres	- Low provision to need ratio -- For every 1000 patients in need of surgery, 44 will be operated on Deprivation - In order to move to middle deprivation quintile, hospitals in most deprived quintile need to perform 24 additional surgeries per 1000 patients - Patients in most deprived quintile had lower need to provision ratios than those in least deprived quintile (95% C.I: 0.30–0.33) - Patients in most deprived quintile had 70% lower provision to need ratios than those in least deprived quintile Geographical/Rurality - Need to provision ratios lowest in north England, West Midlands and London - Highest ratios in south England (except London) - People in more rural areas (village/isolated) had highest need to provision ratios – longer road travel times also had greater provision - Town and fringe areas had lowest need to provision ratios Gender - Men had lower need to provision ratios compared to women, receiving 8% more surgeries (95% C.I: 1.05–1.10) Hospital effects - Higher volume of surgeries, orthopaedic training centre status, more orthopaedic consultants and more operating theatres were associated with higher need to provision ratio Ethnicity - No effect on access seen with ethnic mix of patient residence
[30]	2001/2 financial year	n/a	Waiting time until hip replacement from initial specialist referral to surgery (calculated from HES data)	- Age - Gender - Area-based deprivation (2004 IMD) - Number of diagnoses at hospital admission - Primary diagnosis - Hospital fixed effects	Deprivation - Least deprived patients (educationally) wait 12.8 –13.6% less than patients from bottom 3 deprivation quintiles - Most deprived patients (income) wait 7.5% longer than patients from the least deprived quintile Age - Patients over 75 years wait 17-30% less than patients aged 45-54 Gender - Male patients wait 3.5% longer than women Primary diagnosis - Patients with rheumatoid arthritis or osteonecrosis experience shorter waiting times than arthrosis patients: 27% and 45-53% less respectively Hospital effects - 14% of waiting time variation are as a result of hospital-level differences
[35]	2002 to 2009	Hip replacement rates standardised (per area and per year) to national age-sex specific hip replacement rates for specific year	Admissions data from NHS hospitals	- Age - Gender - Area-based deprivation	- Mean rate of hip replacement for 2009: 20.2 per 10,000 Deprivation - Patients from least deprived quintile receive 5.68 more hip replacements per 10,000 than the most deprived quintile (35% more likely) (95% CI: 5.18–6.18) - Relative increase in age-sex adjusted inequality ratio from 1.23 to 1.35 between 2002 and 2009 (12% increase) (CI 1.25–1.45)
[36]	SMR data: April 1998 to March 2008 ISD data: financial year 2002/3 and 2007/8	n/a	Admissions and data on patient waiting times from SMR	- Age - Gender - Area-based deprivation (Scottish IMD 2006)	- Number of hip replacements increased by 42% from 4095 in 2002-2003 to 5829 in 2007-2008 - Proportion of NHS-funded surgeries undertaken in private hospitals rose from 1.1% in 2002-2003 to 2.9% in 2007-2008 Deprivation - Most deprived quintile had least amount of hip replacements compared to least deprived - 82.8 per 100,000 (95% C.I: 79.2–86.3) for most deprived in 1998-2003 versus 95.3 per 100,000 (95% C.I:91.5–99.0) for least deprived - No significant change in socioeconomic inequality from 1998 to 2008 (*p* = 0.108) Geographical -Significant reduction in geographical inequality (*p* < 0.001) from 1998 to 2008

NOTE: NHS = National Health Service; SASH = Somerset and Avon Survey of Health; ELSA = English Longitudinal Study of Ageing; HES = Hospital Episode Statistics; IMD = Indices of Multiple Deprivation; ECHO = European Collaboration for Healthcare Optimisation; SMR = Scottish Morbidity Records; ISD = Information Services Division; CI = confidence interval

In terms of waiting times, Laudicella et al. [30] showed that the most educated patients’ (IMD Q1) waiting times for surgery were 16.5% shorter than for less educated patients (Q2-5). The same trend applied to patient income as patients with the lowest income (Q5) waited 7.5% longer than patients with the highest income (Q1). From 1997 to 2000, Cooper et al. [27] reported that each decreasing quintile below Q1 (Carstairs index) was associated with an additional 1-2 week wait for surgery (*p* < 0.001). Despite this, by 2007, they reported almost uniformly distributed waiting times across the deprivation quintiles. Cooper et al. [27] was the only access-related study to report an overall decrease in waiting time and SES inequality from 1997 to 2007. Judge et al. [1] reported that people aged 60-64 received more surgeries relative to need compared to those aged 50-59. Those aged over 85 also received less surgery (CI: 0.65–0.72). Laudicella et al. [30] also reported that patients aged 75 years and older waited 17-30% less than patients aged 45-54. These patients were also more likely to experience a greater number of disabilities. Cooper et al. [27] reported that men received 8% more surgeries relative to need compared to women (CI: 1.05–1.10). Despite this, Laudicella et al. [30] reported male patients as having to wait 3.5% longer compared to female patients. Judge et al. [1] reported that the ethnic mix of patients’ area of residence (represented as non-White people versus White people) did not affect access to hip replacement surgery.

### Provision of hip replacement surgery

Table 7 shows the results for the three included studies that explored the provision of hip replacement surgery, as determined by the rate of surgery [4, 31, 32].. From 1991 to 2001, Cookson et al. [31] reported that the rate of hip replacement in England rose from 160 per 100,000 to 184 per 100,000. An increase in provision was observed among more deprived patients, with utilisation rate ratios for the most-deprived quintile (Townsend index Q5) rising from 0.804 to 0.843. The increase in surgical rate required for the rate of surgery in the most-deprived patients to match the rate in the least-deprived patients fell from 41 to 27%. In patients aged 50 to 59 years, Judge et al. [32] found the most deprived had the greatest surgical provision. Despite this, an inverse effect was seen in patients over 85; provision decreased with increasing deprivation. Women received greater provision across all age groups than men; however, the effect was weakest in the oldest and youngest age groups. Geography influenced gender variation; men in the London Borough of Lambeth received 28% less provision than women, compared to men in Wansbeck, north-east England, who received 20% more provision than women. However, Smith et al. [4] reported little difference in provision between men and women. Cookson et al. [31] reported that in both 1991 and 2001, surgical provision was lower than expected for patients in the lowest third of SES. Smith et al. [4] also reported fewer surgical procedures were performed on Black and Asian patients than expected. Ethnic minority patients were younger and had greater physiological ASA (American Society of Anaesthesiologists) fitness grade, but were likely to live in more deprived areas. Surgeries performed on Black patients were more likely to use uncemented hip prostheses instead of cemented prostheses. Despite this, Judge et al. [32] reported no differences in procedure related to patient ethnicity. Smith et al. [4] also reported that Black and Asian patients were more likely to receive hip replacements due to osteonecrosis, rheumatoid arthritis and congenital dysplasia compared to white patients. Surgical provision was greater in hospitals with more operating theatres and higher surgical rates. Despite this, hospitals with greater numbers of consultants, specifically anaesthetic consultants, had lower rates of provision. In terms of rurality, non-urban patients experienced greater surgical provision, as did patients living further from the hospital.

**Table 7:** Results of studies exploring surgical provision

Ref.	Study timeframe	Estimate of surgical provision	Socio-demographic domains	Conclusions
[31]	Financial year 1991/2 and financial year 2001/2	Admissions data from HES	- Age - Gender - Area-based **deprivation** (Townsend deprivation Z-score for 1991 and 2001 census data)	- 1991 hip replacement rate for adults over 44 years: 160 per 100.000, 2001 hip replacement rate: 184 per 100,000 Deprivation - Decrease in inequality from 1991 to 2001 with increase in usage by most deprived patients (standard utilisation rate: 0.804 to 0.843) and decrease in usage for least deprived patients (1.135 to 1.075) - To bring usage levels of most deprived quintile to the level of least deprived quintile, an increase of use of 41% was required in 1991, falling to 27% in 2001 - Utilisation ratio between most deprived and least deprived patients fell from 1.41 in 1991 (95% CI: 1.36–1.47) to 1.27 in 2001 (95% CI: 1.36–1.47)
[32]	Financial year 2002/3	Admissions data from HES	- Age - Gender - Area level deprivation (IMD 2004) - Rurality of residence - Ethnic mix of residence - Hospital variables (number of hip operations, orthopaedic training centre status, rate of consultants, operating theatres and bed occupancy rates	Deprivation - Weak evidence for a trend in relationship between SES and surgical provision Age - In patients aged 50-59, the more deprived patients received more provision, however effects weaken with increasing age – patients aged over 85 had opposite association Gender - Women had greater provision however association was weakest in the oldest and youngest age cohorts – strongest effect in urban areas Ethnicity - No association was found between provision and ethnicity Rurality - Non-urban dwelling patients had greater provision as did those living further away from hospitals Hospital effects - Higher volume of surgeries, more consultants, more anaesthetic consultants and more operating theatres were associated with greater provision Geographical - Certain variables such as gender varied geographically – in some areas men received greater provision, in other areas men receive worse provision
[4]	April 2003 to December 2012	Admissions data from HES	- Age - Gender - Area based deprivation (IMD 2010) - Ethnicity - Primary diagnosis - BMI - ASA score - Quality of life (EQ-5D score)	- Provision of surgery for Black and Asian population lower than expected: Odds ratio for Black patients = 0.33 (95% CI: 0.31–0.35), Odds ratio for Asian patients = 0.20 (95% CI: 0.19–0.21) Type of surgery - Black patients were more likely to receive uncemented prostheses compared to cemented prostheses, in all age groups: Odds ratio = 1.43 (95% CI: 1.11–1.84) Deprivation - Ethnic minority patients were younger and lived in areas of greater deprivation than White patients - Ethnic minority patient had better surgical fitness however (lower ASA grade) Gender - Ratio of expected versus observed surgeries was similar in men and women Primary condition - Osteoarthritis was most common primary condition for all ethnicities - Black and Asian patients more likely to have osteonecrosis, inflammatory arthritis or congenital dysplasia as their primary condition, as well as ‘other reasons’

NOTE: HES = Hospital Episode Statistics; CI = confidence intervals; IMD = Indices of Multiple Deprivation; SES = Socioeconomic status; NJR = National Joint Registry; BMI = Body mass index; ASA = American Society of Anaesthesiologists Physical Status Classification System; EQ-5D = EuroQol-5D

### Surgical outcome of hip replacement surgery

Table 8 shows the results for the five included studies that explored surgical outcomes [28, 33, 37–39]. Preoperatively, Clement et al. [39] reported that the most-deprived patients (Carstairs deprivation category) [DEPCAT] (7 out of 7) scored 5.8 points lower than the least-deprived (DEPCAT 1) on a scale of self-reported hip condition (Oxford Hip Score [OHS]). Neuburger et al. [33] also reported a mean OHS 4.0 points lower in the most-deprived patients (IMD Q5) versus the least-deprived (Q1). Jenkins et al. [37] reported that more deprived patients (SIMD Q5 versus Q1) had worse self-reported hip condition (Harris Hip Score (HHS)) pre-surgery (CI: 0.88–6.82), at 6 months after surgery (CI: 1.92–8.14), and 18 months after surgery (CI: 0.74–8.35). At 6 months after surgery, Neuburger et al. [33] reported a mean OHS 5.0 points lower for the most-deprived patients (Q5) versus the least-deprived. The most-deprived patients were 3.2% more likely to report no improvement in their hip condition after surgery and were also more likely to report a decline in condition. Cookson and Laudicella [38] reported that the most-deprived patients remained in hospital after surgery 6% longer in 2001, falling to 2% longer by 2007. At 18 months after surgery, Jenkins et al. [37] reported significantly worse mental and physical wellbeing in more deprived patients (Short-Form 36-point survey [SF-36] physical: *p* < 0.001; SF-36 mental: p < 0.001). Neuburger et al. [33] identified that 33% of patients living in the most-deprived areas reported poor general health compared to 18% in the least-deprived areas. More deprived patients also had more comorbidities, except for cancer. Cookson and Laudicella [38] reported that patients with seven or more comorbidities stayed in hospital 58% longer than other patients in 2001, increasing to 73% longer by 2007. Clement et al. [39] reported that the comorbidity that predicted no improvement in condition 12 months after surgery was depression. In contrast, Jenkins et al. [37] reported no differences associated with SES and preoperative comorbidities. Despite this, a greater proportion of patients with an ASA status grade I (normal, healthy patient) were in the lowest deprivation quintile (Q5) compared to the highest quintile (Q1) (CI: 1.409–4.044). Another surgical risk identifier, the Physiological and Operative Severity Score for the enumeration of Mortality and Morbidity (POSSUM), was used by Hollowell et al. [28], who showed a modest socioeconomic gradient in POSSUM score, with surgical risk significantly decreasing from deprivation quintile Q5 to Q1 (IMD) (*p* = 0.04). However, no evidence was found between SES and postoperative morbidity. Clement et al. [39] also found no significant association between overall postoperative morbidity and SES but did find a significant association with post-operative hip dislocation in the most-deprived groups (DEPCAT 6 and 7) (*p* < 0.001). No significant difference between patients’ SES and BMI was found by Clement et al. [39] (*p* = 0.05 for no association hypothesis) and Jenkins et al. [37] (*p* = 0.68). Jenkins et al. [37] showed a significantly lower proportion of active smokers in the least-deprived quintile (Q1) compared to the most-deprived (Q5).

**Table 8:** Results of studies exploring surgical outcomes

Ref.	Study timeframe	Estimate of surgical need	Estimate of surgical outcome	Socio-demographic domains	Conclusions
[37]	January 1998 to March 2005	Harris Hip score to determine severity of hip condition and SF-36 to determine patient quality of life ASA score to determine surgical risk Reported patient comorbidities	Harris Hip Score SF-36	- Age - Gender - Area-based deprivation (Scottish IMD 2006) - BMI - Smoking status - Comorbidities - Surgical complications - Mortality - ASA score of presurgical fitness - Quality of life (SF-36 mental and physical)	- 42.6 point improvement in HHS 18 months post-surgery (95% C.I: 41.8–43.4) - No overall change in mean SF-36 mental score 18 months post-surgery (95% C.I: 0.2–2.5) Deprivation - No difference in post-surgical HHS improvement between deprivation quintiles (*p* = 0.069) - The most deprived patients had HHS 3.85 points lower pre-surgery (95% C.I: 0.88–6.82), 5.03 points lower 6-months post-surgery (95% C.I: 1.92–8.14) and 4.55 points lower 18-months post-surgery (95% C.I: 0.74–8.35) than the least deprived patients - Significant difference in physical quality of life between least and most deprived patients: physical SF-36 score was 8.09 points higher (95% C.I: 1.45–14.73) - Mental-health quality of life only improved in the least deprived: mental SF-36 core was 4.7 points higher (95% C.I: 1.5–7.8) - No significant differences in length of stay (*p* = 0.936) - Significantly fewer smokers in the least deprived quintile compared to most deprived (*p* < 0.001) - No significant difference in BMI between quintiles Gender – no significant differences (*p* = 0.238) Comorbidity – no differences
[28]	March 2004 to October 2005	POSSUM score to determine physiological risk factors, condition severity and surgical risk ASA score to determine surgical risk Reported patient comorbidities	POMS survey to assess post-operative morbidity	- Age - Gender - Area-based deprivation (IMD 2004) - White ethnicity - ASA score - Surgery duration - Morbidity	Deprivation - Surgical risk decreased with decreasing deprivation quintile (Q5-Q1) (*p* = 0.04) - 2% increase in predicted surgical risk for most deprived quintile versus least deprived quintile - No evidence for relationship between SES and postoperative morbidity or infectious morbidity - No relationship found between post-surgical length of stay and patient SES - Less deprived patients more likely to be morbidity-free and have left hospital by day 8 post-surgery
[39]	January 2006 to November 2008	Charlson index of comorbidity to assess patient comorbidities Oxford hip score to determine severity of hip condition SF-12 score to determine patient quality of life	Custom patient satisfaction questionnaire Oxford hip score SF-12 score	- Age - Gender - Area-based deprivation (Carstairs index) - BMI - Primary diagnosis - Comorbidities - Surgical complications - 90-day mortality rate - Quality of life (SF-12 score)	Deprivation - No association was found between SES and prevalence of hip replacement (*p* = 0.36) - DEPCATs were significant predictors for mean post-surgical improvement, after adjusting for pre-surgical scores, comorbidity, age, SF-12 score and length of stay (p = 0.001) -Most deprived patients had mean pre-surgery OHS 5.8 points higher than least deprived patients (*p* = 0.001) - Most deprived patients with higher pre-surgery OHS had greater improvement in postoperative score than least deprived patients with lower pre-surgery OHS - Most deprived patients were more likely to suffer dislocation (p < 0.001) and had higher 90-day mortality risk (*p* = 0.02) Age/comorbidities - More deprived patients were younger at time of surgery (p = 0.04) and had more comorbidities (*p* = 0.02) - No association was found between mean Charlson index of comorbidities and SES however (*p* = 0.09) - No association was found between SES and BMI (*p* = 0.5)
[33]	April 2009 to February 2011	Oxford hip score to determine severity of hip condition Patients asked how long their hip problems been present and which comorbidities they have	Oxford hip score ‘Overall, how are the problems now in the (hip/knee) on which you had surgery, compared with before your operation?’	- Age - Gender - Area-level deprivation (IMD 2007) - Non-white ethnicity - Primary diagnosis - Comorbidities - General Health - Hospital fixed effects	Deprivation - Most deprived patients had lower pre-surgery OHS than least deprived: most deprived = 15.7, least deprived =19.7 - Most deprived patients had lower post-surgery OHS than least deprived: most deprived = 34.4, least deprived = 39.4 - More deprived patients reported greater hip-related pain and disability 6 months post-surgery, in addition to poor circulation and depression - 8.2% of most deprived patients reported no improvement post-surgery versus 5.0% of least deprived patients Comorbidities - Patients in more deprived areas had more self-reported comorbidities (except cancer) and poorer overall health (33% poor health in most deprived versus 18% in least deprived) Age/Ethnicity - More deprived patients were more likely younger (below 60 years) and of non-white ethnicity - After adjusting for age, sex, poor pre-surgery health, comorbidities and ethnicity, the association between SES and post-surgery OHS was reduced
[38]	2001 to 2008	Patient comorbidities extracted from HES data	Post-surgical length of stay in hospital from admission to discharge (including treatment for surgical complications)	- Age - Gender - Area-based deprivation (IMD 2004) - Number of diagnoses - Hospital activity	Deprivation - Least deprived patients stay 0.9 days less than the most deprived patients at the same hospital - Most deprived patients stay 6% longer than other patients in 2001-2002, but this fell to 2% longer by 2007-2008 Age - Patients over 85 years stayed 7.74 days longer than patients aged 45-54 - Larger differences between age groups dwarf deprivation gradient in length of stay for age Comorbidities - Patients with 7 or more comorbidities stay 7.18 days longer than patients with one diagnosis - Length of stay for patients with 7+ comorbidities rose from 58% longer in 2001-2002 to 73% by 2007-2008 Hospital effects - Positive hospital-level association – hospitals treated lower SES patients have longer lengths of stay (after adjusting for other patient characteristics)

NOTE: SF-36 = 36-Point Short Form Survey; ASA = American Society of Anaesthesiologists Physical Status Classification System; BMI = Body mass index; HHS = Harris Hip Score; POSSUM = Physiological and Operative Severity Score for the Enumeration of Mortality and Morbidity; POMS = Postoperative Morbidity Survey; IMD = Indices of Multiple Deprivation; CI = confidence interval; SF-12 = 12-Point Short Form Survey; DEPCAT = deprivation category; OHS = Oxford Hip Score; PROM = Patient-Reported Outcome Measure; HES = Hospital Episode Statistics

## Discussion

### Socioeconomic inequalities in hip replacement surgery

Socioeconomic inequality was the most widely measured variable affecting access. In England, the most-deprived patients received 70% lower surgical provision relative to need compared to the least-deprived [1]. One study reported that some lower SES patients reported worse hip condition after surgery [33]. In contrast, a Dutch study [40] found no evidence of educational levels impacting postoperative patient quality of life, as assessed by the SF-36 questionnaire. A Swedish study [41] also investigated education-related deprivation but found no association with postoperative mortality risk, questioning its applicability as a sole indicator of deprivation. Interestingly, a study [42] focusing only on older-age patients (46 to 64 years old) found the only commonly used socioeconomic indicator independently associated with health was income. Education, social class and occupational complexity had no independent effects on health in older-age patients. This is an important consideration for future studies investigating hip replacement surgery as osteoarthritis typically starts at around 50 years of age [43]. However, one study found a stronger relationship between social class and surgical need than English IMD and surgical need [34]. Some study cohorts were not representative of the wider UK population due to fewer patients in more deprived IMD quintiles [2, 33]. This is a notable sampling bias that can reduce the reliability of deprivation indicators such as the IMD. None of the included studies provided evidence that authors implemented controls for measuring deprivation in older age groups. The Income Deprivation Affecting Older People (IDAOPI) is a supplementary index in the English IMD, [44] tailored to assess income deprivation for over-60-year-olds. A study that specifically focused on the income index of IMD, one of seven areas assessed in the IMD to show overall deprivation, did not use the IDAOPI [30], increasing the risk of sampling bias. Both the Carstairs and Townsend deprivation indices use employment as part of their assessment of deprivation [44]. Over the timeframes (1991 to 2008) of the studies that used these indices [27, 31, 39], the UK employment rate of over 65-year-olds was only around 5.5 to 7.3% [45]. With a lower employment rate in more elderly patients, employment is an inadequate indicator of deprivation for hip replacement patients. Furthermore, hip osteoarthritis has been associated with early retirement [46], which suggests the actual employment rate for hip replacement patients is lower than the UK average. Future studies must cautiously consider which measure of socioeconomic deprivation to choose, ensuring that the outcome will be valid for their study’s sociodemographic characteristics.

### Ethnicity-related inequalities in hip replacement surgery

Three studies exploring ethnic mix found no association between access, need or provision of surgery and ethnic mix of patient residence [1, 32, 34]. This contrasts with evidence from the USA, where a study in individuals with federal health insurance showed that Black patients were 30% less likely to undergo hip replacement surgery than White patients, after age and gender-standardisation [47]. Furthermore, Black patients were also shown to have worse preoperative and postoperative pain and function scores [48]. One of the studies reporting no association between ethnic mix and surgical need also reported that while initial data reported an association, this was eliminated by controlling for social class and deprivation [34]. Through an awareness of the intersectionality between ethnicity and SES, studies can explore distinct trends in inequality without conflating the two variables. Ethnicity-related inequalities have been shown to be distinct from SES in a study assessing income-based inequality [47]. In this case, income-based inequality was a more suitable indicator for assessing SES as it allows for individual-level analysis. Ethnic mix and IMD are area-level ecological measures that are not able to show causation or be extrapolated to the individual level. It is essential to recognise this ecological bias, as in order for ethnic inequality to be distinctly explored, both ethnicity and socioeconomic status need to be individually assessed.

Two studies explored distinct ethnic minority groups [2, 4]. One study found Black and Asian patients are more likely to suffer from rheumatoid arthritis and osteonecrosis as their primary condition before hip replacement [4]. Both conditions were also associated with shorter waiting times due to their increased severity over osteoarthritis [30]. When only osteoarthritis patients were assessed, more severe hip condition was shown in Black and South Asian patients [2] – however the minimal clinically important differences (MCID) in OHS were not met [49]. Nevertheless, the increased urgency of surgery in patients with osteonecrosis and rheumatoid arthritis [30] presents a potential confounding variable. Also, the majority of studies that controlled for primary diagnosis only removed cancer or trauma-related hip replacements [1,31, 34,,39], which are known to disproportionately affect lower social classes [35]. To reduce the impact of primary diagnosis as a confounder, future studies might focus on specific preoperative diagnoses. However, it must be noted that there is strong evidence that the quality of ethnicity data reported by individual hospitals varies [50]. Studies investigating differences between individual ethnic minorities should consider this potential for misclassification error in their sensitivity analyses. Furthermore, cross-sectional studies exploring trends over multiple hospitals should ensure other inter- and intra-hospital variables are not responsible for observed patient-level trends [30, 33, 38]. In standard regression analysis, hypothesised unequal surgical provision in ethnic minorities could be explored using rate of surgery as a dependent variable and the ethnic diversity of hospital staff as a key predictor. Even if this investigation were able to prove the original hypothesis, unobserved variables such as differences in primary diagnosis introduce omitted variable bias [51]. Hospital fixed-effects models include hospital dummies in the regression analysis to control for observed and unobserved variables, such as primary diagnosis, diminishing potential omitted variable bias [52]. Studies performing regression analyses should consider using a hospital fixed-effects model to mitigate the impact of omitted variable bias.

### Geographical inequalities in hip replacement surgery

Scottish geographical inequality in access to hip replacement surgery declined from 1998 to 2008 [36], however, England has a distinct north-south divide in surgical access [1]. The higher the need-to-provision ratio, the greater the gap between high surgical need and low surgical provision, with ratios being the highest in southern England (except Greater London) and lowest in northern England. Variations exist within this divide, with domains such as male gender having pockets of higher provision in low need-to-provision ratio areas [32]. This is despite evidence showing men had lower surgical need and provision compared to women [1, 32, 34]. Studies have suggested a ‘postcode lottery’ effect might be responsible [36]. This effect describes certain areas that provide greater provision due to discrepancies in resource allocation by local CCGs. No relationship between rurality and need was found by one study [34]; however, need-to-provision ratios were higher in rural areas [1]. This contrasts with findings that urban hospitals with greater surgical capacity have greater surgical provision [1, 32]. However, higher provision ratios for rural patients provide a potential explanation for studies that found associations between longer travel times for treatment and increased provision [1, 32]. Differences in rurality also affect area-level deprivation measures, such as the IMD. A US study showed that area-level deprivation measures significantly disagreed with individual-level deprivation measures in rural-urban mix areas [53]. A new poverty index is currently under development by the Department of Work and Pensions as an individual-level alternative to deprivation indices [54]. It is hoped future studies may utilise this index to provide more reliable data on healthcare inequalities across the UK. One method the UK government has used to attempt to reduce geographic inequality was creating independent sector treatment centres in 2002 [55]. These are private hospitals contracted by the NHS to conduct elective procedures. One included study [36] reported a reduction in NHS-funded private hip replacements in Scotland from 2008 to 2011 from 8.3 to 0.8%. However, in England, private hospitals conducted 30% of all NHS-funded hip replacements in 2017-18 [56]. Increases in NHS-funded private surgeries have been associated with the diversion of funds from the NHS to the private sector [57]. In Scotland this has resulted in significantly reduced direct NHS surgical provision (*P* < 0.01), and a wider socioeconomic gap in provision, measured using SIMD. While provision inequity between socioeconomic groups is still apparent in the UK, evidence shows the gap has fallen over time in England [27, 31, 38]. Consequently, an increase in NHS-funded private surgeries threatens to weaken past improvements in socioeconomic and geographical inequality. With fears regarding the privatisation of the NHS increasing [58], researchers should investigate the relationship between the proportion of NHS-funded surgeries and socioeconomic inequality in the UK.

### Lifestyle and comorbidity inequalities in hip replacement surgery

Increasing numbers of CCGs in the UK have begun implementing rationing measures for smokers and obese patients [59]. Concerns have been raised over such measures, with arguments that obesity and smoking are linked to lower SES and therefore, rationing would disproportionately affect lower SES patients. Significantly fewer current smokers were observed in more deprived quintiles (*p* < 0.001) [37]. Despite this, two studies [37, 39] investigating surgical outcomes showed obesity as having no relationship to SES. In addition, evidence suggests that other preoperative comorbidities, which are more common in lower SES patients [2], are not perceived as an access barrier to hip replacement surgery [60]. Nevertheless, it is vital that regardless of the involvement of SES, patients with higher BMI scores do not face discrimination. Recent evidence [61] from the US shows that while obesity is linked to a greater risk of surgical complication, 6-month postoperative SF-36 physical wellbeing scores were similar between obese and non-obese hip replacement patients. Furthermore, a study [62] investigating smokers, ex-smokers and non-smokers found no clinically important difference in postoperative patient-reported outcome measures (PROMs) between groups, although greater mortality and complication risk were observed. This evidence shows that the basis behind smoking and obesity-related rationing measures is weaker than suggested, and such measures should be reviewed to ensure they do not unnecessarily discriminate. As of 2009, the NHS has required preoperative and postoperative PROMs to be collected for all hip replacement surgery patients [63]; however, completing these questionnaires is not mandatory. Younger, deprived, non-white men who live alone and have poorer quality of life have been linked to higher non-response rates. Caution should be exercised when interpreting outcomes based solely on PROMs, as non-response bias may cause misrepresentation of the groups that face the greatest healthcare inequality. Another issue faced when assessing PROMs is what change in score can be considered clinically meaningful. For the OHS, the MCID was calculated to be a 5-point increase or decrease [49]. Of the four [2, 29, 33, 39] included studies that used the OHS, two [33, 39] achieved the MCID necessary to prove their association between deprivation and surgical outcome. The other two studies [2, 29] failed to reach the MCID, harming the validity of reported access inequalities in age, gender, ethnicity and deprivation despite their statistical significance. Despite this, one study [29] still reported their findings as statistically highly significant, as the reported *p*-value was < 0.001. One included study [37] used the HHS; however, scores failed to achieve the MCID of between a 7- and 10-point [64] change. No MCID values could be found for the New Zealand score used by two included studies. Researchers must ensure that the clinical importance of findings is not purely based on statistical significance, and relevant MCIDs are used for the intervention being assessed and PROM used.

### Limitations of review

Only one study had Welsh data and no studies had Northern Irish data. Excluding large samples of the UK population introduces selection bias, as the missing population data may have changed the pattern of inequalities described. Consequently, a narrower approach individually focussing on England or Scotland may have been more suitable. While a lack of research may be responsible for the lack of Welsh and Northern Irish data, it is also possible that geographical search criteria may have been imprecise. A custom UK geographical filter was used for the MEDLINE database search [65]; it was only effective for that specific database (Ovid). The simplified filter used for other databases may have excluded relevant studies. Development of an automated, internet or software-based tool to remap search syntax between different databases would allow the custom filter to work in other databases. The risk of bias is challenging to assess for observational studies and is further limited by the included studies’ data heterogeneity. Different checklist questions may have different weightings on study bias, making interpretation of summary scores challenging. There is a need for a standardised methodology to assess the risk of bias in observational studies. This methodology must be easy to apply and allow identification of individual risks of bias, whilst facilitating quick comparison between the overall risks of bias in different studies.

## Conclusion

This review summarises the available literature on access inequalities in hip replacement surgery for the UK. While the heterogeneity of study outcomes and methodology made drawing conclusive evidence challenging, it is clear that access inequality is a major issue in the UK. Potential inequalities in pre-surgical patient consultation were not explored in the included studies. Patient diagnosis and referral to surgery may be impacted by implicit biases present in practitioners, such as an ethnic bias in pain evaluation for Black patients [66]. Despite the unknown prevalence of such ethnic biases, their potential impact signals the importance of increasing workforce diversity, in addition to mandatory implicit bias training for NHS staff. This review demonstrates that there is a shortage of studies that assist in understanding the relationship between sociodemographic or socioeconomic variables and health inequalities. There is a need for bigger studies with more variables based on routinely gathered healthcare data. These studies need to be complemented by PROMs and ethnographic approaches to gather patient narratives. This will assist the development of better services to address inequalities. Given ongoing protests for racial equality and the impact of the COVID-19 pandemic, now is a crucial time to tackle gaps in equality and prevent their growth.

## Data Availability

The datasets used and/or analysed during the current study are available from the corresponding author on reasonable request.

## Appendix 1

**Table 9:** Full database search strategy for included studies

	Search terms		
PICo tool^†^	MEDLINE (Ovid)	PubMed	Web of Science
**Population**	exp Arthroplasty, Replacement, Hip/ OR exp. Hip Joint/ OR exp. Hip Prosthesis OR exp. Osteoarthritis, Hip/	((Arthroplasty, Replacement, Hip [mh]) OR (Hip Joint [mh]) OR (Hip Prosthesis [mh]) OR (Osteoarthritis, Hip [mh]))	TS = hip arthroplasty OR TS=Hip Joint OR TS=Hip Prosthesis OR TS=Hip Osteoarthritis
**Interest**	exp Socioeconomic Factors/ OR exp. Social Class/ OR exp. Ethnic Groups/ OR exp. Minority Groups OR Demography.mp	((Socioeconomic Factors [mh]) OR (Social Class [mh]) OR (Ethnic Groups [mh]) OR (Demography [TIAB]) OR (Minority Groups [mh]))	TS=Socioeconomic Factors OR TS=Social Class OR TS = Ethnic Groups OR TS = Minority Groups OR TS=Demography
**Context I**	exp Health Services Accessibility/ OR exp. “Health Services Needs and Demand”/ OR exp. Social Justice/ OR exp. Health Care Reform/ OR exp. Delivery of Health Care/ OR exp. Health Planning/ OR exp. Health Policy/ OR exp. Healthcare Disparities OR exp. Health Status Disparities OR Health Services.mp OR (equalit$ OR inequalit$ or equit$ or inequit$).tw	((Health Services Accessibility [mh] OR (Health Services Needs and Demand [mh]) OR (Social Justice [mh]) OR (Health Care Reform [mh]) OR (Delivery of Health Care [mh]) OR (Health Planning [mh]) OR (Health Policy [mh]) OR (Healthcare Disparities [mh]) OR (Health Status Disparities [mh]) OR (Health Services [TIAB]) OR (equalit*) OR (inequalit*) OR (equit*) OR (inequit*))	TS=Health Services Accessibility OR TS=Health Services Needs OR TS = Health Services Demands OR TS=Social Justice OR TS=Health Care Reform OR TS=Delivery of Health Care OR TS=Health Planning OR TS=Health Policy OR TS=Healthcare Disparities OR TS=Health Status Disparities OR TS=Health Services OR TS = equalit* OR TS = inequalit* OR TS = equit* OR TS = inequit*
**Context II**	Custom geographical filter developed by Ayiku et al. [65]	(United Kingdom OR England or Wales OR Scotland OR Great Britain OR GB OR UK)	(ALL = (United Kingdom OR England or Wales OR Scotland OR Great Britain OR GB OR UK))

^†^To complete the search, PICO tools were combined as follows: Population AND Interest AND Context I AND Context II

- ‘exp’ refers to an exploded search whereby more niche MeSH keywords relating to the wider concept were captured

- ‘.mp’ refers to a multi-purpose search whereby the specific term is searched for in several fields of the article, including the title and abstract

- ‘.tw’ refers to a text word search whereby the specific term is searched for only in the title and abstract for added specificity

- ‘[mh]’ refers to a MeSH specific search which ensures the terms are searched as MeSH-specific keywords only

- ‘[TIAB]’ refers to title and abstract search whereby the specific term is searched for only in the title and abstract

- ‘TS=’ refers to a topic search whereby the specific term is searched for in several fields of the article, including the title and abstract

‘$’ and ‘*’ are truncation symbols allowing for a variety of word-endings to be captured.

## Abbreviations

ASA: American Society of Anaesthesiologists Physical Status Classification System
BMI: Body mass index
CCG: Clinical Commissioning Group
DEPCAT: Deprivation category
EQ-5D: EuroQol-5D quality of life score
HHS: Harris Hip Score
HES: Health Episode Statistics
IDAOPI: Income Deprivation Affecting Older People Index
IMD: Indices of Multiple Deprivation
MeSH: Medical Subject Headings
MCID: Minimal Clinically Important Difference
NHS: National Health Service
OECD: Organisation for Economic Co-operation and Development
OHS: Oxford Hip Score
PROM: Patient-reported outcome measure
POSSUM: Physiological and Operative Severity Score for the Enumeration of Mortality and Morbidity
PICO: Population, Intervention, Control, Outcome model
PICo: Population, Phenomena of Interest, Context model
PRISMA: Preferred Reporting Items for Systematic Review and Meta-Analyses
SIMD: Scottish Indices of Multiple Deprivation
SES: Socioeconomic status
SF-36: 36-Item Short Form Survey
